# Serum glial fibrillary acidic protein as a biomarker of brain injury in premature neonates

**DOI:** 10.17305/bjbms.2021.6205

**Published:** 2021-07-19

**Authors:** Dimitra Metallinou, Grigorios Karampas, Georgia Nyktari, Nicoletta Iacovidou, Katerina Lykeridou, Demetrios Rizos

**Affiliations:** 1 Department of Midwifery, University of West Attica, Athens, Greece; 2 2nd Department of Obstetrics and Gynecology, Medical School, National and Kapodistrian University of Athens, “Aretaieio” University Hospital, Athens, Greece; 3 Neonatal Intensive Care Unit, “Gaia” Maternity Hospital, Athens, Greece; 4 Neonatal Department, Medical School, National and Kapodistrian University of Athens, “Aretaieio” University Hospital, Athens, Greece; 5 Hormone Laboratory, Medical School, National and Kapodistrian University of Athens, “Aretaieio” University Hospital, Athens, Greece

**Keywords:** Brain injury, glial fibrillary acidic protein, premature neonates, intraventricular hemorrhage, periventricular leukomalacia

## Abstract

Neonatal brain injury (NBI) is a serious adverse outcome of prematurity. Early detection of high risk premature neonates to develop NBI is not currently feasible. The predictive value of many biomarkers has been tested, but none is used in clinical practice. The purpose of this study was to determine the levels and predictive value of serum glial fibrillary acidic protein (GFAP) in a prospective longitudinal case–control study during the first 3 days of life in premature neonates (<34 weeks of gestation) that later developed either intraventricular hemorrhage or periventricular leukomalacia. Each case (n=29) was matched according to birth weight and gestational age to one neonate with normal head ultrasound scans. No significant differences in GFAP levels were observed between the groups. Nevertheless, neonates with brain injury presented more frequently with GFAP levels above the lowest detection limit (0.056 ng/ml) and this trend was significantly different during all 3 days. Thus, the effectiveness of GFAP as an early biomarker of NBI in premature neonates seems to be limited.

## INTRODUCTION

Preterm birth (<37 weeks of gestation) is a significant worldwide public health issue with an estimated global rate of 10.6% for the year 2014 with identified data points from 107 countries [1]. Consequences of prematurity are numerous, with the neonatal brain injury (NBI) being one of the most severe ones [2].

The underlying mechanisms of NBI involve an initial insult to the vulnerable fetal brain that is usually either hypoxic–ischemic, hemorrhagic, or infectious in nature. Intraventricular hemorrhage (IVH), periventricular leukomalacia (PVL), and hypoxic-ischemic encephalopathy (HIE) are the most common subtypes of NBI, which can affect neonates born at any gestational age (GA). However, neonates born <32 weeks of gestation are more prone to IVH and PVL, while neonates born >35 weeks to HIE [3]. As a consequence, the initial injury activates a cascade of events leading to further brain damage which increases the risk for serious long-term neurodevelopmental impairment, including motor, cognitive, neurologic, and sensory disability [2].

Although noteworthy progress has been made in the management of preterm neonates, the rates of neonatal morbidity and adverse neurodevelopmental outcomes remain high, underlining the need for early, and individualized therapeutic intervention to prevent severe brain injury [3]. Despite the ongoing research, there is currently no available effective prognostic model used in clinical practice, which may provide early detection of neonates at high risk to develop NBI [3,4]. At present, the identification of high risk premature neonates is based mainly on general clinical characteristics such as birth weight <1500 g, GA <28 weeks and perinatal factors associated with brain injury, such as fetal growth restriction or chorioamnionitis [2,4]. In an effort to provide early therapeutic interventions, on the one hand, and prognostic data on survival or density of residual deficits, on the other hand, a number of brain injury biomarkers are under evaluation since clinical but mostly radiological signs remain silent during the first days of life [4,5].

Of the biomarkers associated with brain injury, preliminary research on glial fibrillary acidic protein (GFAP) appears to be promising in the early recognition of NBI in premature neonates [5,6]. GFAP is a brain-specific cytoskeletal intermediate filament protein with a molecular mass between 40 and 53 kDA, which is localized predominantly in astroglial cells and is released as a consequence of brain injury and astrogliosis [6]. Stewart et al. [6] demonstrated that levels of circulating GFAP on days 1-4 of life are significantly elevated in preterm neonates that later on developed PVL and that even among neonates with IVH, GFAP could identify which ones were at higher risk for the later development of PVL. Serum GFAP has also been reported by Ennen et al. [7]. to be significantly elevated in neonates with GA between 36 and 41 weeks with HIE receiving hypothermia therapy when compared with controls.

Thus, identification of premature neonates who are at risk of developing NBI in the early neonatal period with the use of one or more biomarkers could provide the clinicians with the potential for early intervention. For instance, therapeutic hypothermia in late preterm neonates and brain-focused care, as well as neuroprotective medication in early preterm neonates could probably improve the neurodevelopmental outcomes [8-12]. Yet, there is limited data available regarding the use of GFAP as a biomarker for the early detection of NBI and long-term neurologic outcome, especially in preterm neonates [6]. Furthermore, there is no available study in premature neonates that provides evidence on serum GFAP levels and its predictive value comparing GFAP with the levels and predictive value of other biomarkers, such as S100B, in the same study population and this makes our study of great interest [13].

The purpose of this study was to determine whether serum GFAP levels measured within the first 3 days of life differ between premature neonates (<34 weeks) (a) with and without NBI, (b) with IVH and those with PVL, and (c) with different grades of NBI as well as (d) to evaluate the predictive value of serum GFAP during the first 3 days of life to early identify high-risk premature neonates that will either develop NBI or will be complicated with a severe adverse neonatal outcome such as death or seizures/hypertonia.

### Definitions of NBI

#### IVH

IVH typically initiates in the periventricular germinal matrix, which is particularly vulnerable to hemorrhage in premature neonates mostly in the first 48 h of life [14]. The classic grading system of IVH was initially described by Papile et al. [15]. Findings are graded on a scale from I to IV [16] and it is estimated that it affects 15-20% of the neonates born <32 weeks [2,15-17].

### PVL

PVL is defined as injury to the deep cerebral white matter that can be seen in two characteristic patterns: (a) Focal necrosis with loss of all cellular elements in periventricular white matter and (b) diffuse lesion in cerebral white matter [18]. It is the most common type of brain injury in premature neonates, often associated with or considered as a direct consequence of IVH, but also seen in the absence of IVH. Developmental outcomes for neonates with PVL are related to the grade and location of parenchymal involvement [19]. According to Romero-Guzman et al. [18], prevalence is estimated under 28 weeks at 39.6%, under 32 weeks at 27.4%, and under 37 weeks at 7.3% and it is classified on a scale from I to IV [20].

## MATERIALS AND METHODS

This is an Institutional Review Board (IRB) approved prospective longitudinal case–control study of live born premature (<34 weeks) neonates, born at a single tertiary hospital, who were admitted to the Neonatal Intensive Care Unit (NICU) between November 2016 and March 2018 (“Aretaieio” University Hospital - IRB R.No: B-216/13-10-2016/APPROVAL NUMBER-ID: KM140657). The study is part of a wider research protocol on the levels and predictive value of brain injury biomarkers in premature neonates with and without NBI and was carried out according to “ICMJE Recommendations for the Protection of Research Participants” [13]. All procedures were in accordance with the Declaration of Helsinki.

Inclusion criteria were (a) prematurity (<34 weeks) and (b) NBI in the form of either PVL or IVH for the case group. Neonates with major congenital, genetic, or chromosomal abnormalities as well as other types of NBI, such as HIE, were excluded from the study. Only neonates whose parents gave their written informed consent were included in the study.

All neonates were admitted to NICU right after delivery. According to the NICU’s protocol, on admission routine laboratory investigation included complete blood count (CBC), blood culture, and C-reactive protein (CRP). CBC and CRP should be additionally assessed in all premature neonates on the 2^nd^ and 3^rd^ day of life. Any unused quantity of serum was then used for the measurement of GFAP. Blood was collected from peripheral or umbilical vessels and the residual serum was aliquoted and stored at −35°C until assayed.

NBI was classified at discharge taking into account head ultrasound scans (HUS) and the neonates were allocated in the case or control group. HUS followed the European Standards of Care for Newborn Health (ESCNH) [21] and were all performed in the NICU and evaluated by the Consultant Paediatric Radiologist of the Hospital. HUS through the cranial fontanels is considered as the gold standard for the diagnosis of NBI, allowing rapid bedside evaluation of the neonatal brain [22-24]. Until now, there is no universally accepted protocol for HUS screening in preterm neonates [21,24]. According to the protocol of ESCNH [21], a HUS should be performed in preterm neonates on days 1, 3, 7, 14, 21, and 28 at 6 weeks and at term equivalent age (TEA) if GA is >28 weeks. If GA is <28 weeks, a HUS should be performed on days 1, 3, 7, 14, 21, and 28 then for every 2 weeks until the 34^th^ week GA and at TEA. Finally, a HUS should be intensified in case of abnormalities or after episodes of clinical deterioration (e.g., unexplained anemia, neurological symptoms, surgery, HIE, central nervous system infection, and metabolic disease).

Medical records were reviewed by the study personnel to identify relevant maternal and neonatal data regarding clinical and laboratory perinatal factors that could be of interest to either influence or predict NBI. Before any statistical analysis accuracy of data collection was double checked by the study personnel. Coding of all participants (mothers and neonates) was automatically created by the database used, to preserve anonymization/deidentification.

High-risk pregnancy was defined according to international standards and guidelines including preeclampsia [25], oligohydramnios [26], hypothyroidism [27], gestational diabetes mellitus [28], chorioamnionitis [29], fetal growth restriction [30], and pathological Doppler [31].

Determination of GFAP concentrations was performed with one of the most sensitive commercially available kits (GFAP - MBS2701011, GFAP, ELISA) from MyBioSource, USA. According to the kit’s inserts, the lowest detection limit was 0.056 ng/ml and the precision, as estimated by the total CV (%), was <10%. Values <0.056 ng/mL were reported as zero.

### Statistical Analysis

Statistical analysis was crosschecked and performed by the research team with the use of the commercially available software package: IBM SPSS statistics version 23 (IBM Corporation, Somers, NY 10589, USA). As there were no available studies on the levels of serum GFAP in the general population of premature neonates complicated with the development of NBI, sample size calculation for this wider research protocol was based on the levels of S100B, which is considered as the “gold standard” of NBI biomarkers [13]. Clinical characteristics and laboratory findings of women and neonates included in the study were compared in an effort to assure the success of the matching and to specify dissimilarities between the two groups. Pearson’s Chi-square test (*X*^2^) was performed for comparisons of qualitative data. One-Sample Kolmogorov–Smirnov test was done to control the normality of the distribution of GFAP and the rest of the quantitative parameters. Based on this analysis, either parametric t-test or Mann–Whitney U-test was used to compare GFAP concentration and the other quantitative parameters between the groups. Kruskal–Wallis test was applied to compare levels of GFAP within groups. Control and neonates with either PVL or IVH were included in a subgroup analysis, so as to investigate if GFAP levels varied in different types of NBI. Further subgroup analysis was made to compare GFAP levels in the five deceased neonates of the case group with the levels of control neonates and the rest of the cases, on purpose to identify if GFAP’s levels are altered in case of imminent death during the early neonatal period. Finally, the value of serum GFAP to predict NBI in the first 3 days of life was examined through multivariate logistic regression analysis setting as dependent variable either (a) the development or not of NBI during hospitalization in NICU or (b) the presence or not of Grade II-IV IVH complicated by seizures/hypertonia or death, and as independent variables the levels of serum GFAP and S100B [13] in the same study population during the first 3 days of life. A probability level of less or equal to 0.05 was considered significant.

## RESULTS

In this wide research protocol ninety-six (n=96) neonates fulfilled the inclusion criteria and were finally included in the study [13]. Sixty-five (n=65) of these neonates did not develop NBI while the rest thirty-one (n=31) were complicated with a type of NBI. From the latter, seventeen neonates developed PVL (n=17), twelve IVH (n=12), and two HIE which were excluded from the study. Consequently, the case group consisted of twenty-nine (29) neonates. Sixteen (n=16) neonates from the PVL group were diagnosed with Grade I unilateral or bilateral PVL and one with Grade II unilateral PVL. From the IVH group (n=12), four (n=4) neonates were diagnosed with Grade I unilateral or bilateral IVH and eight (n=8) with Grade II-IV unilateral or bilateral IVH. Six (n=6) neonates of the IVH group had seizures during hospitalization and three (n=3) finally died. The rest three (n=3), apart from seizures, also developed hypertonia. Totally, five (n=5) neonates died, all of which were diagnosed with IVH.

From the sixty-five neonates with normal HUS, twenty-nine were selected to constitute the control group. Matching between cases (n=29) and controls (n=29) was conducted manually in a 1:1 fashion, taking into account closeness of GA (within 1 week) and birth weight. The mean and standard deviation (SD) GA for control neonates and cases were 29.8 ± 2.5 weeks and 29.6 ± 3.0 weeks while birth weight at admission was 1302 ± 429 gr and 1225 ± 475 gr, respectively (Tables 1 and 2).

**TABLE 1:**
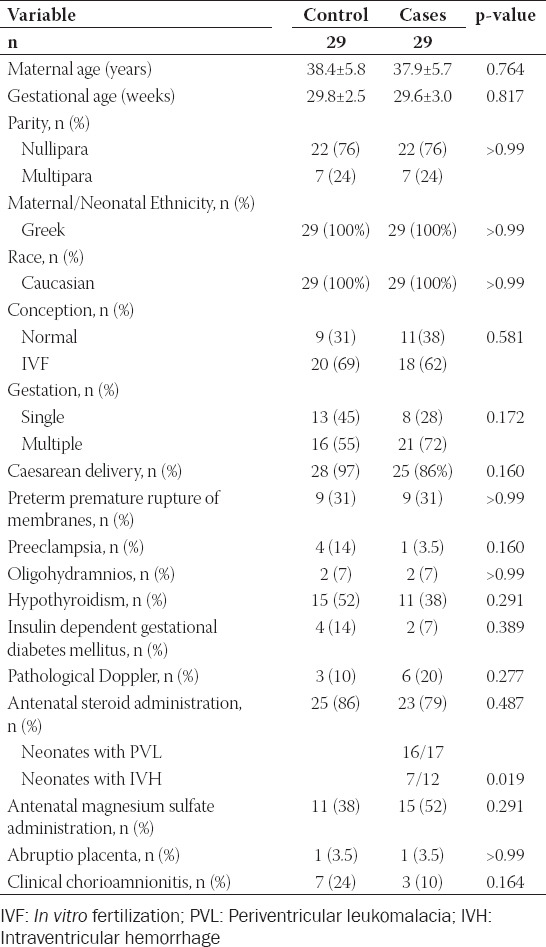
Maternal demographic and clinical characteristics of control and cases neonates

**TABLE 2:**
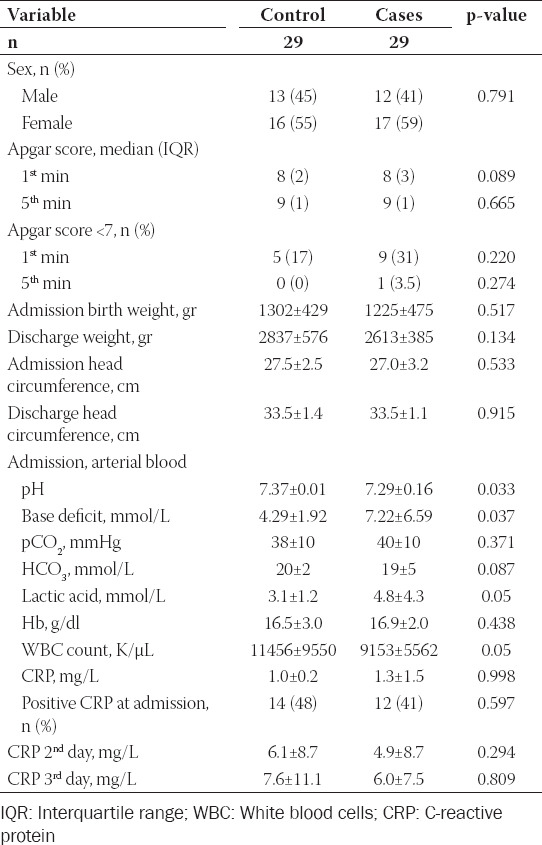
Neonatal demographic - clinical characteristics and laboratory findings of control and cases neonates

Comparison between cases and control neonates for maternal demographic and clinical characteristics identified no differences except for the antenatal use of corticosteroids between women whose neonates developed IVH and those that developed PVL (Table 1).

Neonatal demographic – clinical characteristics and laboratory findings between the case and control group are presented in Table 2. Neonates in the case group had significantly lower admission pH and white blood cells count when compared to control neonates while admission base deficit and concentration of lactic acid were higher in neonates in the case group. Moreover, no difference was observed on therapeutic interventions in neonates between the two groups (Table 3). Finally, regarding neonatal outcomes (Table 4), necrotizing enterocolitis was more frequent in control neonates while seizures and death were more frequent in the case group.

**TABLE 3:**
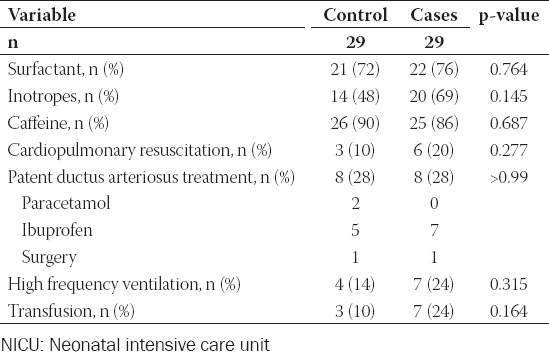
Therapeutic interventions of control and cases neonates

**TABLE 4:**
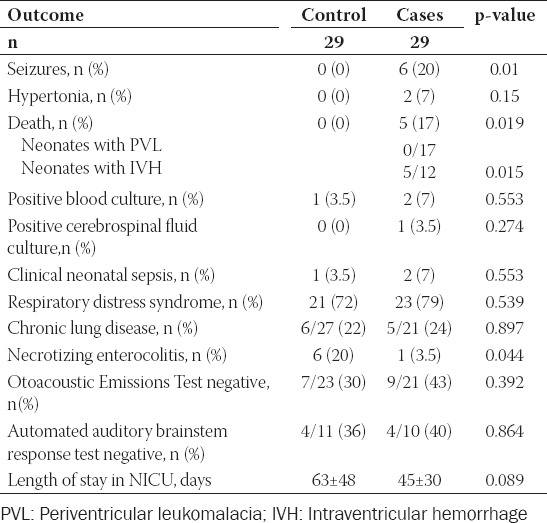
Neonatal outcomes of control and cases neonates

Serum GFAP levels were available for comparison at 85/87 (97.7%) of the desired time points for both case and control group. Missing data were due to insufficient serum after routine investigation had been performed.

**FIGURE 1:**
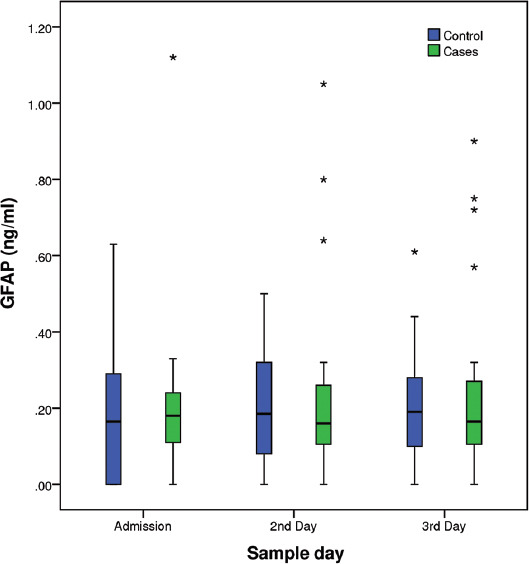
Concentration of serum Glial Fibrillary Acidic Protein (ng/mL) in neonates with and without brain injury during the first 3 days of life. Box plots (horizontal line: median; box: 25-75% percentiles; whiskers: min–max; asterisk: outliers).

Mean ± SD and Median – Interquartile Range of GFAP levels are presented in Table 5. No difference was observed within groups during the first 3 days of life. Moreover, GFAP levels did not differ significantly between control neonates with and without necrotizing enterocolitis. Although mean GFAP concentration (ng/ml) was higher in the case group in all days, this difference was not significant (Table 5, Figure 1).

**TABLE 5:**
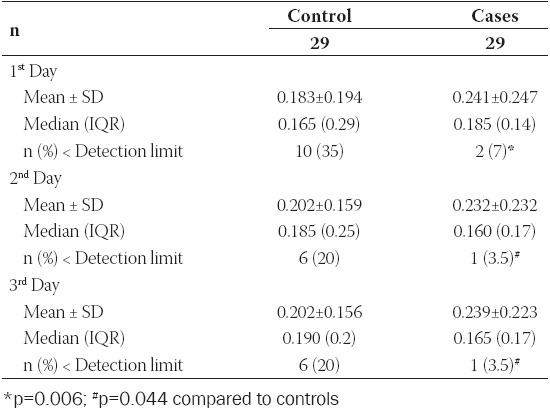
Mean ± Standard Deviation (SD), Median (Interquartile Range, IQR) and frequency of concentrations of Glial Fibrillary Acidic Protein (ng/ml) below the lowest detection limit in neonates with and without brain injury during the first 3 days of life

It is worth mentioning though, that neonates in the case group presented more frequently GFAP levels above the kit’s lowest detection limit (0.056 ng/ml) and these percentages were significantly different during the first 3 days of life (p<0.05, Pearson’s Chi-square test) (Table 5). Moreover, as shown in Figure 1, neonates in the case group presented more frequently very high levels of GFAP (outliers) but these measurements were not necessarily associated with neonates that died.

Further subgroup analysis among control neonates and neonates with either IVH or PVL revealed no significant difference on the levels of GFAP (results not shown). Notably, when the five neonates that died in the case group were compared to either control or the rest of the neonates with NBI, no significant difference was observed during the first 3 days of life (results not shown).

Interestingly, additional subgroup analysis between neonates with PVL and IVH showed that neonates whose mothers received antenatal corticosteroids (Table 1) developed more frequently PVL instead of IVH and had significantly lower probability of neonatal death during hospitalization (Table 4).

Finally, the multivariable logistic regression analysis including as independent variables serum GFAP and S100B levels during the first 3 days of life confirmed that the predictive value of serum GFAP, regarding either the development of NBI or a severe adverse neonatal outcome such as II-IV grade IVH complicated by seizures/hypertonia or death, is limited, as it did not reach statistical significance (results not shown).

## DISCUSSION

At present, there is no model or biomarker that can detect premature neonates at high risk for developing NBI [32-34]. Head ultrasound imaging is considered as the gold standard for the diagnosis but not the prediction of NBI in neonates, especially premature ones that will develop either PVL or IVH [22-24]. Moreover, MRI has been shown to identify moderate-to-severe cerebral white matter injury that can predict adverse neurodevelopmental outcome but again its predictive value regarding NBI in the first days of life is limited [21,35,36]. Early detection of premature neonates that will later on in life develop NBI is crucial, as early therapeutic interventions might moderate neurodevelopmental defects. Numerous biomarkers have been investigated regarding their prognostic value for NBI, but data on the efficacy of GFAP in preterm neonates remain limited.

In the present study, we demonstrate that levels of serum GFAP on days 1-3 of life are elevated in preterm neonates which will later develop NBI. While elevated the difference was not statistically significant. Our findings differ to these by Stewart et al. [6] who detected a significant difference between normal and neonates with NBI. However, the case group in Stewart’s study consisted of either very low birth weight (VLBW) (<1500 g) or LBW neonates (1500-2500 g) “with suspected neurologic morbidity at birth, which included prolonged hypotonia or seizures,” while the case group in our study was more representative of the general population of premature neonates as no weight limit or neurologic morbidity was set as inclusion criteria. Furthermore, many neonates in Stewart’s study developed both PVL and IVH and as mentioned in the same study “GFAP was significantly increased in neonates with both IVH and PVL on days 2-4 of life” when compared to neonates with IVH only. Consequently, when combined, PVL and IVH can elevate cumulatively the levels of GFAP contributing to higher levels in the case group. Contrarily, in our study, the case group consisted of neonates with either PVL or IVH, but not both, representing a well separated study population in terms of subsequent pathology. Nevertheless, as in the study by Stewart et al., we identified a clear trend as GFAP was detected more frequently below the lowest detection limit (0.056 ng/ml) in the control group compared to neonates with NBI.

Our findings are also consistent with the observation that neither neonates that died (n=5) nor those with II-IV grade IVH showed significantly higher GFAP concentrations when compared to neonates of either the control or case group during the first 3 days of life (results not shown). Although the levels of GFAP were more elevated in these neonates when compared to controls, it seems that GFAP is either of limited value in the prognosis of NBI in the general population of premature neonates or not as powerful as other biomarkers, such as S100B [37,38]. The latter is considered as one of the most effective biomarkers to predict severe types of NBI in premature neonates according to a number of previous studies, including ours [13]. More specifically, in our previous study for a cutoff value of 10.51 ng/ml, serum S100B on the 1^st^ day of life performed an excellent sensitivity of 100% and specificity of 93.9% to predict severe adverse neonatal outcome such as death or IVH of II-V grade complicated with seizures and/or hypertonia.

The fact that we have investigated both GFAP and S100B in the same study population is an important strength of our study as no previous study provides evidence on the prognostic value of GFAP when directly compared to the most well studied biomarker of NBI in premature neonates. Another strength of our study is the prospective longitudinal methodology, which allows the repetitive evaluation of GFAP levels. Following that methodology, the absence of significant difference between control and case neonates was confirmed during the first 3 days of life which is the most important period for the early detection of premature neonates at risk to develop NBI.

While not a primary aim of this study, we observed that antenatal corticosteroids, specifically betamethasone, have a neuroprotective effect. In both our study and the study by Stewart et al. [6] neonates whose mothers received antenatal steroids were less likely to develop IVH and had significantly lower rates of mortality.

Finally, our study has some limitations as well. A main limitation is the low number of neonates in both groups which could explain the fact that differences on serum GFAP between the two studied groups did not reach statistical significance. Nonetheless, in our previous study with the same study population, differences on serum S100B levels were significant already from the 1^st^ day of life, indicating that differences on GFAP levels are limited [13]. Moreover, even though we used one of the most sensitive commercial kits for serum GFAP, many neonates in the control group presented GFAP levels below the lowest detection limit, which indicates that future studies should consider using a more sensitive method to measure serum GFAP levels, and consequently determine its predictive value regarding NBI in premature neonates more accurately.

## CONCLUSION

We report that serum GFAP levels in premature neonates (<34 weeks) that develop NBI do not differ significantly to the levels of neonates without NBI during the first 3 days of life. Consequently, its effectiveness as an early predictive biomarker of NBI in the general population of premature neonates is probably limited. However, premature neonates without NBI had more often GFAP levels below the lowest detection limit. Therefore, a more sensitive detection method for serum GFAP in the future might highlight its predictive role in the early identification of NBI in the general population of premature neonates.
